# Multicentric and multifocal *versus* unifocal breast cancer: differences in the expression of E-cadherin suggest differences in tumor biology

**DOI:** 10.1186/1471-2407-13-361

**Published:** 2013-07-26

**Authors:** Tobias Weissenbacher, Eva Hirte, Christina Kuhn, Wolfgang Janni, Doris Mayr, Uwe Karsten, Brigitte Rack, Klaus Friese, Udo Jeschke, Sabine Heublein, Darius Dian, Nina Ditsch

**Affiliations:** 1 Frauenklinik, Klinikum der Ludwig-Maximilians-Universität, Innenstadt, München, Germany; 2 Frauenklinik, Heinrich-Heine-Universität, Düsseldorf, Germany; 3 Pathologisches Institut, Ludwig-Maximilians-Universität, München, Germany; 4 Frauenklinik, Klinikum der Ludwig-Maximilians-Universität, Groβhadern, München, Germany; 5 Glycotope GmbH, Berlin, Germany; 6 Department of Gynecology and Obstetrics, Campus Innenstadt Ludwig-Maximilian-University Munich, Maistr. 11, Munich D-80337, Germany

**Keywords:** Breast cancer, MUC-1, Multicentric, Multifocal, Tumor biology, E-cadherin, β-catenin

## Abstract

**Background:**

The aim of this study was to evaluate the expression of the cell adhesion-related glycoproteins MUC-1, β-catenin and E-cadherin in multicentric/multifocal breast cancer in comparison to unifocal disease in order to identify potential differences in the biology of these tumor types.

**Methods:**

A retrospective analysis was performed on the expression of MUC1, β-catenin and E-cadherin by immunohistochemistry on tumor tissues of a series of 112 breast cancer patients (total collective) treated in Munich between 2000 and 2002. By matched-pair analysis, 46 patients were entered into two comparable groups of 23 patients after categorizing them as having multicentric/multifocal or unifocal breast cancer. Matching criteria were tumor size, histology grade and lymph node status; based on these criteria, patients were distributed equally between the two groups (p = 1.000 each). Data were analyzed with the Kruskal-Wallis and the Mann–Whitney tests.

**Results:**

In the matched groups, we found a significantly down-regulated expression of E-cadherin in multicentric/multifocal breast cancer compared to unifocal disease (p = 0.024). The total collective showed even higher significance with a value of p < 0.0001. In contrast, no significant differences were observed in the expression of β-catenin between multicentric/multifocal and unifocal tumors (p = 0.636 and p = 0.914, respectively). When comparing the expression of MUC1, E-cadherin and β-catenin within the unifocal group, we found a significant positive correlation between E-cadherin and β-catenin (p = 0.003). In the multicentric/multifocal group we observed, in contrast to the unifocal group, a significant decrease of MUC1 expression with increased grading (p = 0.027).

**Conclusion:**

This study demonstrates that multicentric/multifocal and unifocal breast cancers with identical TNM-staging clearly differ in the expression level of E-cadherin. We suggest that the down-regulation of E-cadherin in multicentric/multifocal breast cancer is causally connected with the worse prognosis of this tumor type.

## Background

Tumor-node-metastasis (TNM) staging has been the standard method for breast cancer classification for more than fifty years. During this time, however, the classification procedure has changed substantially. In 2003, the 6th edition of the TNM classification was established [1-3]. The T category has maintained its prognostic relevance throughout these changes [3]. The prognosis of breast cancer patients depends on two different types of factors: tumor size as a time-dependent marker of tumor biology, and biological factors (i.e., histological grade) which represent tumor aggressiveness [4]. Other prognostic factors include the estrogen and progesterone receptor status as well as the relative number of mitotic figures (MF/10HPF) [5,6]. Treatment plans are following worldwide prevailing suggestions, including those of the TNM system. However, the TNM classification has changed, and treatment recommendations and the treatments themselves have been modified. Breast-conserving treatment, once a controversial issue, is now an established alternative to modified radical mastectomy for surgically manageable breast cancer.

In a recent study we have demonstrated that focality is an independent prognostic factor by comparing multicentric/multifocal and unifocal breast cancer [7]. Therefore, additional biological factors seem to play an important but not well understood role in multicentric/multifocal breast cancers.

The above-mentioned established prognostic factors [4,8,9] as well as potential new factors, such as the E-cadherin-related transcriptional repressor Snail or the c-Jun activation domain-binding protein-1 (Jab1), are multifunctional signaling proteins. The E-cadherin/catenin complex is known to be a potent inhibitor of cancer progression [10-13].

The disconnection of cell-cell adhesions is a fundamental step in the progression of cancer and metastasis that is mediated by a variety of membrane proteins. The transmembrane protein E-cadherin, which is responsible for calcium-dependent cell adhesions, is a widely studied tumor suppressor. It is expressed predominantly in epithelial cells, and its extracellular region has a Ca^2+^-dependent homophilic adhesion function. Loss of E-cadherin has been reported to induce epithelial-mesenchymal transition in several cancers [14-16].

Epithelial mucin-1 (MUC1) is a complex transmembrane glycoprotein. The larger, heavily glycosylated domain of the MUC molecule is extracellularly expressed [17]. MUC1 exerts a number of different functions [18-23]. MUC1 undergoes characteristic modifications of its glycosylation and cellular localization during malignant transformation [24]. Many monoclonal antibodies have been developed to MUC1 [17]. A novel antibody, PankoMab, was developed against a tumor-associated epitope of MUC1 [19]. In a previous paper, PankoMab was examined in patients with breast cancer in comparison with two other known antibodies. PankoMab was unique to the effect that its staining was correlated with the estrogen receptor expression [20].

The glycoprotein β-catenin interacts with both E-cadherin and MUC1. The interaction between MUC1 and E-cadherin is mediated by β-catenin-binding and interrupts E-cadherin-mediated cell-cell adhesions. Signal transduction through β-catenin (the so-called Wnt/β-catenin signaling pathway) has already been thoroughly investigated [21]. This signal transduction regulates the expression of a number of genes essential for cell differentiation and proliferation. Alterations in this pathway are implicated in diseases such as cancer [22].

The aim of this study was to compare the expression of MUC1, E-cadherin and β-catenin in multicentric/multifocal tumors with their expression in unifocal tumors of identical tumor size according to TNM staging in order to detect potential differences.

## Methods

### Patients

Two groups were framed and investigated. Based on a consecutive patient cohort consisting of 112 patients documented and surgically treated for primary breast cancer between 2000 and 2002 at the Department of Gynecology of the University Hospital in Munich-Innenstadt, 57 unifocal breast cancer patients and 55 patients with multicentric/multifocal disease formed our total collective (TC). From the same patient cohort, two equivalent groups of 23 breast cancer patients with multicentric/multifocal vs. unifocal tumors were selected using a matched paired analysis (MG) (see Statistical Analysis section below). The Institutional Review Board of the Ludwig Maximilians University Munich, Germany, approved the study and all the patients gave informed consent.

Unifocality versus multicentricity/multifocality were determined by clinical examination, ultrasound and X-ray. In addition, in a few cases nuclear magnetic resonance imaging (NMRI), galactography or pneumocystography was performed if necessary. These techniques were used in a few cases, in which additional information regarding focality was necessary. Moreover, those cases which failed to confirm multicentricity/multifocality with respect to the final histological examination were excluded.

Data were contemporaneously gathered for the unifocal and multicentric/multifocal tumors. To be eligible, patients were required to be free of disease, and they must have been treated at the study site at the time of primary diagnosis of resectable breast cancer. The tumor stage at primary diagnosis was classified according to the UICC TNM classification [23]. Tumor grading by WHO (Nottingham grading respectively to Elston & Ellis modification of Bloom-Richardson grading [25] was used, and match criteria were tumor size, histology grade and lymph node status, all of which were equally distributed between the two groups (p = 1.0). The total collective was not matched. We used this group to validate the results of the matched group.

### Surgical treatment

The primary surgical treatment consisted of either breast conservation or modified radical mastectomy. Routine axillary dissections were performed on levels I and II lymph nodes, while level III lymph nodes were only excised in cases expressing macroscopic metastatic lesions of the lower levels. For the diagnosis of lymph node metastasis, single embedded lymph nodes were screened at up to three levels.

The guidelines for chemotherapy and cytostatic regimes changed substantially also within the observation time of the study. Therefore the authors did not include oncological treatment details.

### Immunohistochemistry

Immunohistochemistry was performed using a combination of pressure cooker heating for antigen retrieval and the standard streptavidin-biotin-peroxidase complex with the use of the mouse IgG-Vectastain Elite ABC kit (Vector Laboratories, Burlingame, CA, USA). Table 1 lists the mouse monoclonal antibodies used for these experiments.

**Table 1:** Antibodies employed

**Antigen**	**Antibody/clone**	**Isotype**	**Dilution**	**Source**
E-cadherin	HECD-1	Mouse IgG1	1:80	Merck, Darmstadt, Germany
β-catenin	polyclonal	Rabbit IgG	1:100	Diagnostic BioSystems, Pleasanton, CA, USA
MUC1	mPankoMab	Mouse IgG1	1:550	Glycotope, Berlin, Germany

Formalin-fixed paraffin embedded tissue sections were dewaxed using xylol for 15 min, rehydrated in an descending series of alcohols (100%, 96%, and 70%), and subjected to epitope retrieval for 5 min in a pressure cooker using sodium citrate buffer (pH 6.0). After cooling, sections were washed twice in PBS. Endogenous peroxidase activity was quenched by immersion in 3% hydrogen peroxide in methanol for 20 min. Non-specific binding of the primary antibodies was blocked by pretreatment of the sections with diluted normal serum (10 ml PBS containing 150 μl horse serum; Vector Laboratories, Servion, Switzerland) for 20 min. Sections were then incubated with the primary antibodies at room temperature for 60 min. After washing with PBS, sections were incubated in diluted biotinylated secondary antiserum (10 ml PBS containing 50 μl horse serum; Vector Laboratories) for 30 min at room temperature. After incubation with the avidin-biotin peroxidase complex (diluted in 10 ml PBS, Vector Laboratories) for 30 min and repeated washing steps with PBS, visualization was performed with DAB substrate (Dako, Glostrup, Denmark) for 2 min. Sections were counterstained with Mayer‘s hematoxylin and dehydrated in an ascending series of alcohols (50–98%), followed by xylol. Finally, sections were embedded, but mounted and covered. Negative controls were performed by replacing the primary antibody with normal horse serum. Immunohistochemical staining was performed using an appropriate positive control.

The intensity and distribution patterns of specific immunohistochemical staining were evaluated using the semi-quantitative immuno-reactive score (IRS). This score was calculated by multiplying the staining intensity (graded as 0 = no, 1 = weak, 2 = moderate and 3 = strong staining) with the percentage of positively stained cells (0 = no staining, 1 = <10% of cells, 2 = 11-50% of cells, 3 = 51-80% of cells and 4= >81% of cells stained). The slides were examined by two independent observers. Sections were examined using a Leitz microscope (Wetzlar, Germany) with a 3CCD color camera (JVC, Victor Company of Japan, Japan).

### Statistical analysis

Data were entered into the database in a coded fashion. Our total collective of 112 patients included 57 unifocal breast cancer patients and 55 cases of multicentric/multifocal tumors. Because of the uneven distribution of prognostic factors in our original patient group of 46 cases that met the match criteria, a matched pair analysis was performed. A total of 23 pairs of patients, each consisting of one patient with unifocal and one with multicentric/multifocal tumor lesions, were selected according to the highest degree of equivalence in the following hierarchical and sequential order: tumor size at the time of primary diagnosis, histology grading, and lymph node status. Each parameter was required to have a p value > 0.50 to achieve intergroup homogeneity. We deliberately matched patients based on the criteria at the time of primary diagnosis. The computer software ‘Statistical Package for the Social Sciences 15.0’ (SPSS Inc., Chicago, IL, USA) was used to perform statistical analyses. We used Kruskal-Wallis one-way analysis of variance to analyze our data, which is a non-parametric method for testing equality of population medians among groups. It is an extension of the Mann–Whitney U test to 3 or more groups.

For survival analysis median immunoreactivity levels, as determined by the IR-score, of each marker were employed to split the collective into low vs. high expressing cases. The following thresholds were used: E-Cadherin ≥ IRS 8, beta-Catenin (membrane staining) ≥ IRS 8, beta-Catenin (cytoplasma staining) ≥ IRS 4, MUC1 (membrane staining) ≥ IRS 8, MUC1 (cytoplasma staining) ≥ IRS 1. Kaplan-Meier survival curves were drawn to compare survival times of uni- vs. multifocal/-centric tumors and of high vs low expressing cases, respectively. Differences in overall and relapse-free survival were tested for significance by applying the chi-square statistic of the log rank test.

P values below 0.05 were considered significant.

## Results

All matching criteria (tumor size, histology grade and lymph node status) were equally distributed between the two groups (p = 1.0).

No significant difference was observed between the two groups in terms of age (p = 0.104 in the matched group and p = 0.533 in the total collective) or menopausal status (MG: p = 0.291 and TC: p = 0.503). Regarding histological types of tumors, the total collective (TC) demonstrated a statistically significant difference with p = 0.003 (see below), whereas no significant difference was found in the matched group (p = 0.120). Table 2 shows the primary patient characteristics of both groups.

**Table 2:** Patient characteristics

**Total collective**			
	**Multicentric/multifocal (%)**	**Unifocal (%)**	**P-value**
**Number of patients**	55	57	
**Age**	60.6	58.9	**.533**
**Lymph node Metastases**			**.150**
Absent (N0)	27 (50.0)	35 (62.5)	
1-3 axillary LNM (pN1bi)	4 (7.4)	7 (12.5)	
1-3 axillary LNM (pN1biii)	18 (33.3)	8 (14.3)	
1-3 axillary LNM (pN1biv)	0	3 (5.4)	
4-9 axillary LNM (pN2)	1 (1.9)	1 (1.8)	
Unknown (pNx)	5 (9.1)	3 (5.3)	
**Histological Type**			**.003**
Ductal	35 (66)	39 (69.6)	
Lobular	11 (20.8)	3 (5.4)	
Ductal-lobular	4 (7.5)	3 (5.4)	
Mucinous	1 (1.9)	2 (3.6)	
Medullary	1 (1.9)	4 (7.1)	
Micropapillary	1 (1.9)	2 (3.6)	
Tubulary	0	3 (5.4)	
Not specified	2 (3.6)	1 (1.8)	
**Menopausal Status**			**.503**
Premenopausal	13 (47.9)	16 (37.5)	
Postmenopausal	37 (52.1)	36 (62.5)	
**Matched Group**			
	**Multicentric/multifocal (%)**	**Unifocal (%)**	**P-value**
**Number of patients**	23	23	
**Age**	57	68	**.104**
**Histological Type**			**.120**
Ductal	16 (69.6)	15 (65.2)	
Lobular	5 (21.7)	3 (13.0)	
Ductal-lobular	2 (8.7)	1 (4.3)	
Medullary	0	1 (4.3)	
Micropapillary	0	2 (8.7)	
Not specified	0	1 (4.3)	
**Menopausal Status**			**.291**
Premenopausal	4 (18.8)	6 (26.1)	
Perimenopausal	0	1 (4.3)	
Postmenopausal	18 (81.8)	14 (60.9)	
Unknown	1 (4.3)	2 (8.7)	

Looking at the total collective, 55 patients were included in the multicentric/multifocal group and 57 in the unifocal group. This group was not matched, so statistical analysis was performed according the matching criteria of tumor size, lymph node status and histopathological grading. Tumor size (p = 0.113), lymph node involvement (p = 0.150), and histopathological grading (p = 0.068) did not show any significant correlation with multicentric/multifocal tumors versus unifocal tumors.

According to the histological tumor type, a significant difference was observed in the incidence of invasive lobular cancer in the multicentric/multifocal group in comparison to the unifocal group. Of 14 patients suffering from invasive lobular cancer, 11 had multicentric/multifocal disease, whereas only 3 had unifocal breast cancer. The results were different for invasive ductal tumors; out of 74 patients with invasive ductal cancer, 35 had multicentric/multifocal disease, and 39 had unifocal breast cancer. Looking at the matched group, five patients had lobular multicentric/multifocal breast cancer (21.7%), and three patients (13.6%) had a lobular unifocal disease. Also, ductal carcinomas did not differ significantly. Sixteen patients (69.6%) in the multicentric/multifocal matched group had ductal breast cancer, compared with 15 patients (68.2%) in the unifocal group.

**Figure 1:**
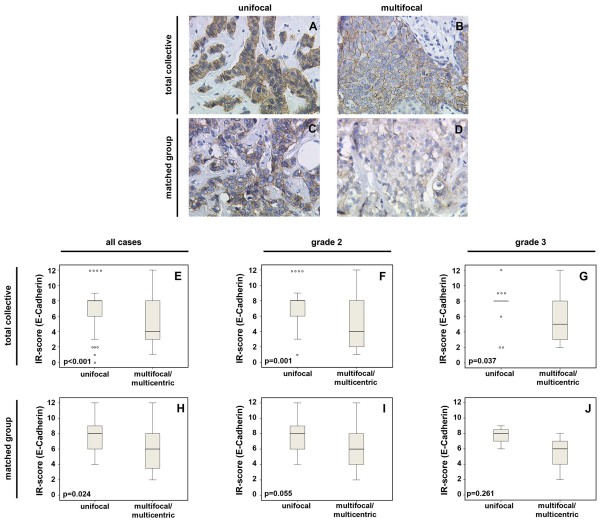
**E-cadherin expression in the total collective (A, B) and in the matched group (C, D) of unifocal (A, C) and multicentric/multifocal (B, D) breast cancer; magnification 25× lens.** Semiquantitative evaluation of staining results (IR score) is presented in box plots **(E-G)** for the total collective and in the box plots **(H-J)** for the matched group with respect to differences between G2 and G3-tumors. The boxes represent the range between the 25th and 75th percentiles with a horizontal line at the median. The bars delineate the 5th and 95th percentiles. The circles indicate values more than 1.5 box lengths away from the median.

Regarding the expression of E-cadherin, lobular cancers were not included in the statistical analysis of the two groups. The total collective examined therefore included 54 unifocal and 44 multicentric/multifocal cancer tissues. Compared to the multicentric/multifocal group, E-cadherin expression was significantly higher in the unifocal group, with a p-value of <0.0001. MG in this case included 32 patients (16 pairs). E-cadherin expression was also significantly higher in the unifocal matched group with p = 0.024 (Figure 1).

Looking at the grading within the total collectivegroup as well as unifocal tumors, G2 (moderately differentiated) tumors exhibited higher E-cadherin expression compared to multifocal tumors (p = 0.001), as did G3 (poorly differentiated) tumors (p = 0.037). The matched pair group underlined these results for G2 tumors and revealed higher E-cadherin expression in unifocal tumors compared with multicentric/multifocal tumors. The p-value was 0.055 for G2 tumors, whereas G3 tumors failed to demonstrate significance with p = 0.261 (Figure 1).

No significant differences in β-catenin expression patterns were observed between multicentric/multifocal and unifocal tumors (p = 0.914) when comparing the total collective, and the difference was also not significant for the matched pairs (p = 0.636). Furthermore, β-catenin expression showed no significant correlation with histology grade within the total collective either for multicentric/multifocal breast cancers (p = 0.564) or for unifocal disease (data not shown, p = 0.635).

**Figure 2:**
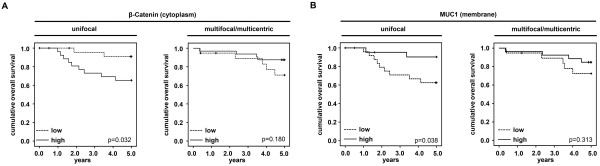
**ß-Catenin and MUC1 expression related to overall survival and focality. A**: Cytoplasmic ß-catenin expression related to the overall survival (OS) in unifocal and multicentric/multifocal tumors. **B**: PankoMab epitope on the membrane related to the overall survival (OS) in unifocal and multicentric/multifocal tumors.

However, the cytoplasm ß-catenin was associated significantly with a reduced overall survival (OS) in unifocal tumors (p = 0.032). Interestingly, no differences were found concerning survival in mulicentric/multifocal tumors (Figure 2A).

The MUC1 expression also failed to demonstrate a significant difference between unifocal and multicentric/multifocal disease in both the MG (p = 0.840) and the TC group (p = 0.183).

**Figure 3:**
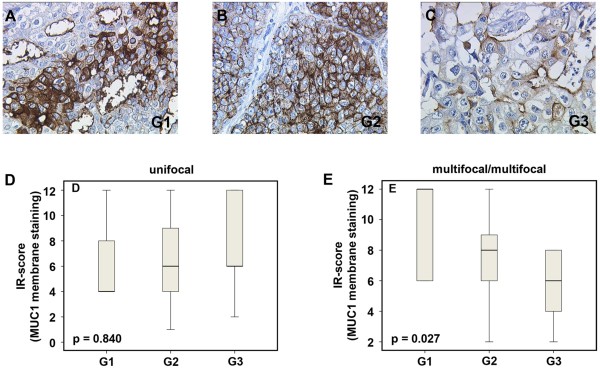
**MUC1 (mPankoMab) membrane expression in the total collective of multicentric/multifocal breast cancer in a G1 tumor (A), a G2 tumor (B), and a G3 tumor (C); magnification 25× lens.** The box plots **(D, E)** present a semiquantitative evaluation of staining results (IR score). The boxes represent the range between the 25th and 75th percentiles with a horizontal line at the median. The bars delineate the 5th and 95th percentiles.

Analyzing differences with respect to histology grade, no differences in MUC1 expression were observed in the total collective among G1, G2 and G3 unifocal tumors (p = 0.840). In contrast, MUC1 expression in multicentric/multifocal tumors was significantly dependent on histology grade (decreasing from G1 to G3 at p = 0.027) (Figure 3).

The PankoMab epitope demonstrated no difference according to the histology grade when looking at the cytoplasm staining. When looking at the overall survival (OS), the PankoMab epitope on the membrane was associated with a better outcome, however only significant in G2 and G3-unifocal tumors **(**Figure 2B).

In other words, less differentiated multicentric/multifocal tumors exhibited partial loss of MUC1 expression.

## Discussion

We investigated in a previous study the prognostic differences between multicentric/multifocal and unifocal breast cancer [7]. In that study, patients were entered by matched-pair analysis into two comparable groups of 288 patients after categorizing them as having multicentrical/multifocal or unifocal breast cancers. Matching criteria were tumor size, histology grade and hormone receptor status, which were equally distributed between both groups (p = 1.000 each). We demonstrated that multicentric/multifocal breast cancer is associated with a worse prognosis compared to unifocal disease with an identical tumor size [7]. However, Vlastots et al. investigated breast cancer patients with early-stage disease and did not find an increased risk of poor outcome with respect to multicentricity. According to the authors, this study supports the current tumor, node, metastasis staging system [26].

On the contrary, Tot et al. also demonstrated recently, that multifocality represents a negative prognostic parameter associated in this study with significantly increased lymphnode metastasis (LNM) [27]. These findings were confirmed by Tot et al. in further studies, that demonstrated multifocality being associated with an increased risk of LNM [28,29].

According to our study-collective of 112 patients, 55 patients were included in the multicentric/multifocal group and 57 in the unifocal group. This total collective was not matched, and statistical analysis was performed according the matching criteria of tumor size, lymph node status and histopathological grading. Our results did however not demonstrate any significant correlation of lymph node metastasis when comparing multicentric/multifocal and unifocal tumors. This result however has to be interpreted in a critical manner to the effect that the total collective however includes patients who were matched according to the lymph nodes status.

However, it remained unclear whether the tendency of breast cancer tumors to metastasize was a reflection of the total tumor load or whether biological differences play a decisive role. The 10-year survival rate was investigated by Boyages et al. who found – especially in tumors > 2 cm – that the aggregate size of every focus should be considered along with other prognostic factors when comparing multifocal and unifocal breast cancer [30].

Aim of this manuscript was, to evaluate differences in tumor biology, that might help explaining the above mentioned differences. Tot et al. investigated multifocal and unifocal breast cancer according to the immunophenotype (estrogen and progesterone receptor expression, HER2 overexpression and expression of basal-like markers, CK5/6, CK14, and epidermal growth factor receptor). The auhors found higher rates of LNM in the multifocal group, interestingly no differences with respect to molecular phenotype [29]. These findings were underlined by Pekar et al. who also demonstrated that diffuse or multifocal distribution of the invasive component is associated with cancer-related death independent of the molecular phenotype [31].

Bassarova et al. [32] investigated the cadherin/catenin immunophenotype of multicentric tumor foci and bilateral breast cancer. They found a greater similarity of the primary tumor to its corresponding metastatic tumor than to the contralateral primary tumor regarding the cadherin/catenin immunophenotype [32]. Although different histological subtypes were examined (pleomorphic lobular, invasive ductal of usual type, atypical medullary carcinomas, mucinous and invasive micro papillary carcinomas), differences in the tumor biology were obvious and could be anticipated. The present study was intended to analyze some of the potential factors involved.

β-catenin is involved in cell-cell adhesions and is a transcriptional regulator in the Wnt signaling pathway [33], furthermore it is consequently involved in the development of human malignancies. Lopez-Knowles et al. [34] investigated immunohistochemically the expression of β-catenin in 292 patients with invasive ductal breast cancers. The authors demonstrated an association between a high cytoplasmic expression of β-catenin and a high tumor grade (p = 0.004) and negative estrogen receptor values (p = 0.005), and the high expression of β-catenin was thus associated with an adverse disease outcome.

We found no differences for the cytoplasmic ß-catenin as well as for the membrane ß-catenin with respect to the grading. Moreover, the cytoplasmic ß-catenin was associated significantly with a reduced OS in unifocal tumors (p = 0.032). Our data suggest a wnt signaling pathway in unifocal tumors. However, this pathway might not play an important role in multicentric/multifocal tumors. Therefore we assume differences in tumor biology between uni- and multifocal tumors according to our results.

Niu et al. described an association between abnormal β-catenin expression, positive lymph node status and high histological grade (p < 0.01) as well as a significant correlation between positive Her2 expression and abnormal β-catenin expression [13]. Therefore, elevated β-catenin expression appears to be linked with worse outcome for the patients. However, differences concerning focality have not been investigated.

Recent research has underlined the importance of E-cadherin with respect to cell adhesion mechanisms. Down-regulation of E-cadherin/catenin-mediated intercellular adhesion is known to be an important step in the acquisition of malignancy and metastasis. According to Baranwal [14], down-regulation of E-cadherin is associated with worse outcome and enhanced aggressiveness of the tumor. Klopp et al. [35] also stated that decreased expression of E-cadherin is associated with breast cancer progression and resistance to therapy. Finally, loss of E-cadherin expression is a hallmark of epithelial-mesenchymal transition (EMT), which is associated with a worse prognosis [16]. In contrast, up regulation of E-cadherin/catenin complex, which acts as a suppressor of tumor progression, has been accomplished with a series of agents, some of which can be used therapeutically [36].

Our finding of a significantly reduced expression of E-cadherin in multicentric/multifocal tumours underline and reinforce our view of a more aggressive behavior of this tumor type. Since loss of E-cadherin is a marker of EMT, it might be worthwhile to examine other EMT markers such as MMPs, which lead to E-cadherin degradation [37], or vimentin in multicentric/multifocal *versus* unifocal breast tumors.

MUC1 is a multifunctional epithelial glycoprotein known to be overexpressed in most epithelial cancers. MUC1 can promote proliferation and metastasis, whereas down regulation of MUC1 expression inhibits cell migration by inducing β-catenin relocation from the nucleus to the cytoplasm and increases E-cadherin/catenin complex formation [38]. In addition, MUC1 is coexpressed and complexed with STAT1 (Khodarev et al. [39]), and it is associated with decreased recurrence-free and overall survival. This may explain why intracellular expression of MUC1 is associated with worse prognosis [40], whereas membrane (or overall) expression of MUC1 is generally correlated with a better outcome [41].

Using the anti-MUC1 antibody mPankoMab, which recognizes a special, tumor-associated MUC1 epitope [19], we previously observed a correlation between MUC1 and the expression of the ER receptor [42]. In the present study, we did not observe differences in MUC1 expression between multicentric/multifocal and unifocal breast cancer (p = 0.183). However, when looking at the histopathological grading, multicentric/multifocal carcinomas showed a statistically significant decrease in staining with increased histology grade (p = 0.027) which was in contrast to the MUC1 expression in unifocal breast cancer of different grade.

According to the cytoplasmic PankoMab-staining no differences were found with respect to the histology grade. When looking at the overall survival (OS) the PankoMab epitope on the membrane was however associated with a better outcome, nevertheless only significant in G2 and G3 unifocal tumors (p = 0.038).

## Conclusions

In summary, differences regarding tumo rbiology are obvious as fore the wnt signaling pathway might play an important role in unifocal tumors and the PankoMab epitope on the membrane associated with a better outcome in G2 and G3 unifocal tumors.

Due to the small collective used for this study, we have not confirmed and extended our earlier results which demonstrated that multicentric/multifocal tumors as compared to unifocal breast tumors correlate with a reduced survival and relapse-free interval (Additional file 1: Figure S1). Instead, we analyzed membrane associated breast cancer markers as molecules to discriminate with respect to focality between both entities. These results indicate that the breast tumor biology differs depending on focality and suggest a tendency for enhanced EMT in multicentric/multifocal breast cancer. Further research is necessary on the tumor biology of multicentric and multifocal tumors.

## Competing interest

Uwe Karsten is an employee of Glycotope GmbH which mad and provided the PankoMab antibody. All other authors declare no competing interest.

## Authors’ contributions

TW designed the study and performed collection, analysis and interpretation of data and drafted the manuscript for publication. EH, CK and UK participated in the design of the study, and were involved in the immunhistochemistry. WJ, SH, ND, BR essentially were involved in the analysis and interpretation of the data and also approved the English. UJ, DD and FK performed participant inclusion, collected samples and contributed substantially to acquisition of data. DD helped substantially to draft the manuscript. All conceived of the study, participated in its design and coordination, helped with data interpretation and drafting of the manuscript. All authors read and approved the final manuscript.

## Pre-publication history

The pre-publication history for this paper can be accessed here:

http://www.biomedcentral.com/1471-2407/13/361/prepub

## Supplementary Material

Additional file 1: Figure S1 Kaplan-Meier survival curves were drawn to compare Overall survival (OS) and relapse free survival (RFS) in unifocal and multicentric/multifocal tumors. Click here for file
