# Supplementary figures and images for: Multicentric and multifocal versus unifocal breast cancer: differences in the expression of E-cadherin suggest differences in tumor biology
Source: BMC Cancer. 2013 Jul 26;13:361. doi: 10.1186/1471-2407-13-361 (PMC3729674; doi:10.1186/1471-2407-13-361)

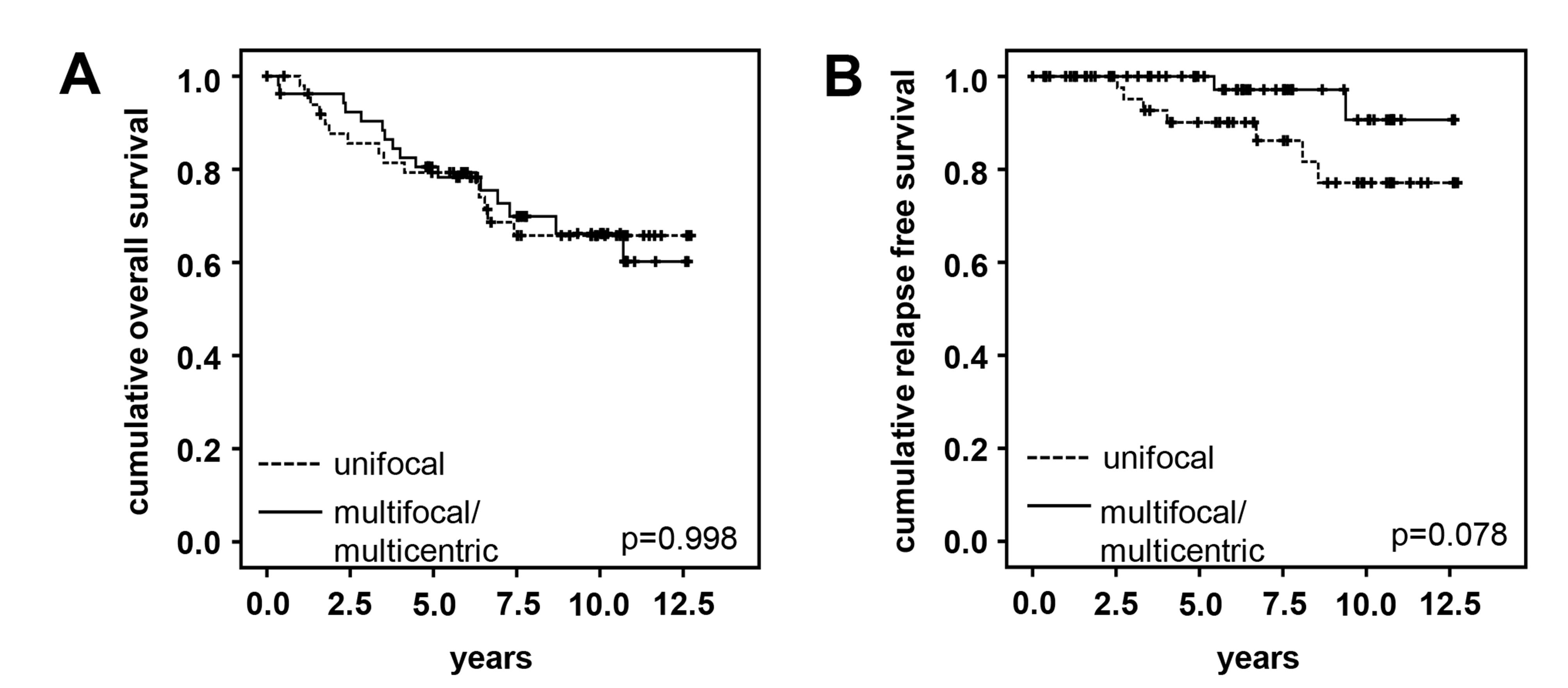

Supplement: Additional file 1: Figure S1 — Kaplan-Meier survival curves were drawn to compare Overall survival (OS) and relapse free survival (RFS) in unifocal and multicentric/multifocal tumors. [file 1471-2407-13-361-S1.jpeg]
